# Identifying novel mechanisms of abdominal aortic aneurysm *via* unbiased proteomics and systems biology

**DOI:** 10.3389/fcvm.2022.889994

**Published:** 2022-08-03

**Authors:** Stephanie Morgan, Lang Ho Lee, Arda Halu, Jessica S. Nicolau, Hideyuki Higashi, Anna H. Ha, Jennifer R. Wen, Alan Daugherty, Peter Libby, Scott J. Cameron, Doran Mix, Elena Aikawa, A. Phillip Owens, Sasha A. Singh, Masanori Aikawa

**Affiliations:** ^1^Cardiovascular Division, Center for Interdisciplinary Cardiovascular Sciences, Brigham and Women's Hospital and Harvard Medical School, Boston, MA, United States; ^2^Channing Division of Network Medicine, Brigham and Women's Hospital and Harvard Medical School, Boston, MA, United States; ^3^Department of Physiology, Saha Cardiovascular Research Center, University of Kentucky, Lexington, KY, United States; ^4^Center for Excellence in Vascular Biology, Brigham and Women's Hospital and Harvard Medical School, Boston, MA, United States; ^5^Department of Cardiovascular Medicine, Section of Vascular Medicine, Heart Vascular and Thoracic Institute, Cleveland Clinic Foundation, Cleveland, OH, United States; ^6^Division of Vascular Surgery, Department of Surgery, University of Rochester School of Medicine, Rochester, NY, United States; ^7^Division of Cardiovascular Health and Disease, Department of Internal Medicine, University of Cincinnati College of Medicine, Cincinnati, OH, United States

**Keywords:** inflammation, thrombosis, network analysis, angiotensin II, elastase, mouse model

## Abstract

**Background:**

Abdominal aortic aneurysm (AAA), characterized by a continued expansion of the aorta, leads to rupture if not surgically repaired. Mice aid the study of disease progression and its underlying mechanisms since sequential studies of aneurysm development are not feasible in humans. The present study used unbiased proteomics and systems biology to understand the molecular relationship between the mouse models of AAA and the human disease.

**Methods and results:**

Aortic tissues of developing and established aneurysms produced by either angiotensin II (AngII) infusion in *Apoe*^−/−^ and *Ldlr*^−/−^ mice or intraluminal elastase incubation in wildtype C57BL/6J mice were examined. Aortas were dissected free and separated into eight anatomical segments for proteomics in comparison to their appropriate controls. High-dimensional proteome cluster analyses identified site-specific protein signatures in the suprarenal segment for AngII-infused mice (159 for *Apoe*^−/−^ and 158 for *Ldlr*^−/−^) and the infrarenal segment for elastase-incubated mice (173). Network analysis revealed a predominance of inflammatory and coagulation factors in developing aneurysms, and a predominance of fibrosis-related pathways in established aneurysms for both models. To further substantiate our discovery platform, proteomics was performed on human infrarenal aortic aneurysm tissues as well as aortic tissue collected from age-matched controls. Protein processing and inflammatory pathways, particularly neutrophil-associated inflammation, dominated the proteome of the human aneurysm abdominal tissue. Aneurysmal tissue from both mouse and human had inflammation, coagulation, and protein processing signatures, but differed in the prevalence of neutrophil-associated pathways, and erythrocyte and oxidative stress-dominated networks in the human aneurysms.

**Conclusions:**

Identifying changes unique to each mouse model will help to contextualize model-specific findings. Focusing on shared proteins between mouse experimental models or between mouse and human tissues may help to better understand the mechanisms for AAA and establish molecular bases for novel therapies.

## Introduction

An aneurysm is characterized by a localized expansion of arterial wall, which may portend rupture (1). Abdominal aortic aneurysms (AAA) occur primarily between the renal arteries and the bifurcation of the iliac arteries (2), affecting up to 8% of men over the age of 65 (3). In addition to male sex, other risks for AAA include smoking, advanced age, previous myocardial infarction, and family history (4). Many AAAs remain asymptomatic and only incidentally undergo detection through abdominal imaging such as ultrasonography, computed tomography angiography, and magnetic resonance imaging (1). Despite many efforts to establish predictive or diagnostic imaging techniques (5), circulating biomarkers (6), or disease features such as intraluminal thrombus (ILT) (7) for indication of rupture-prone aneurysms, none have proven to be sufficiently robust to be implemented in clinical practice. No medical approaches to reducing AAA expansion have been validated, rather, once an aneurysm diameter has reached an established “threshold” [4.5–5.0 cm for women and 5.5 cm for men (8)], the only treatment option involves surgical repair. Better understanding the pathogenesis of AAA may provide a molecular basis for new targeted medical therapies for the prevention of AAA progression and rupture.

Studies of tissues collected from patients undergoing surgical repair have defined the pathological characteristics of AAAs, including loss of extracellular matrix (ECM) integrity, medial degeneration, and inflammation (9). Proteomics-based studies have explored further features of human AAA such as the contribution of the ILT (10), identifying differential markers within the ECM (11), or pursuing potential circulating biomarkers (12). While the reliance on mice to study this disease is convenient, the relevance or extent of overlap between AAA mice and the human condition remains unclear.

Among several types of experimental AAA in mice (13, 14), the most common are provoked by either angiotensin II (AngII) or elastase (15). AngII is infused using a subcutaneously implanted osmotic pump and produces suprarenal AAAs in ≤1 weeks (13). AngII–infusion is usually performed in hypercholesteremic *Apoe*^−/−^ or *Ldlr*^−/−^ mice fed a high fat and cholesterol enriched “Western” diet (16). In the elastase model, either intraluminal elastase perfusion or topical elastase incubation in/on the infrarenal aorta results in immediate arterial dilation with cell death, elastin fragmentation, and inflammation driving further diameter expansion through 2 weeks after surgery (14). Each model recapitulates selected features of human AAA (e.g., inflammation, ECM destruction, luminal dilation, intact intima, and rupture-induced death), but neither covers the full spectrum of pathology. While the AngII model in *Apoe*^−/−^ mice more closely mirrors the human disease in its inclusion of hypercholesterolemia, a relatively chronic disease development, and the heterogeneity of aneurysms between mice, its mortality rate reaches higher (nearly 40%) than in humans (17) and the thrombus occurs extraluminally due to medial rupture resulting in adventitial dissection (17). In the elastase model, surgical intervention and manipulation drives initial inflammation and aortic dilation producing consistent and uniform aneurysms without requirement of genetic alteration. However, the elastase model relies on experienced microsurgical skills and the administration of a foreign substance (i.e., porcine elastase).

Prior studies have explored mechanisms of aneurysm expansion by examination of temporal changes in tissue pathology (18, 19), or similarities between mouse and human tissues (20). The present study uses a systems approach, involving unbiased mass spectrometry-enabled proteomics, high-dimensional bioinformatics, and systems biology, to complete proteome profiling of the three most commonly used mouse models of AAA: systemic AngII infusion into *Apoe*^−/−^ and *Ldlr*^−/−^ mice and localized elastase perfusion into wildtype mice. The initial goal involved identifying both commonality and disparity amongst these three models. To further substantiate such an approach in mice, we performed proteomics in a set of human AAAs which may unify the human condition with each model *via* their common and potentially critical molecular signatures. Ultimately, this study aims to better understand mechanisms that drive AAA, providing molecular bases for new therapies.

## Methods

The Online Data Supplement contains additional detailed methods. Male *Apoe*^−/−^ (*n* = 12) or *Ldlr*^−/−^ (*n* = 13) mice in a C57BL/6J background at 10–12 weeks of age, underwent infusion of AngII *via* a subcutaneously placed osmotic minipump (13). Male wildtype C57BL/6J mice (10–12 weeks of age, *n* = 10) underwent experimental AAA creation with the luminal infusion of porcine pancreatic elastase (14). The aorta was cut into 8 segments (segment 1, aortic arch; segments 2–4, thoracic; segment 5, suprarenal; segment 6 corresponded with renal vessels; segments 7 and 8, infrarenal). Human AAA tissue was obtained from nine patients (4 female and 5 male) aged 65 ± 7.7 years undergoing open aneurysm repair at the University of Rochester Medical Center. The patient criteria for open surgical repair were defined as AAA diameter exceeding 55 mm for males, AAA diameters between 50 and 55 mm for females, rapidly expanding aortic diameters (≥5 mm in 6 months with a minimum diameter of 40 mm) or symptoms attributable to AAA and AAA rupture. Mural thrombi were collected during surgery, along with residual aortic wall fragments, and were immediately flash frozen until protein processing. Control tissue was procured from the infrarenal aortas (5 males and 5 females) of deceased individuals aged 58 ± 6.6 years and rapidly frozen for analysis. Mouse and human tissue was proteolyzed using either a chloroform:methanol extraction strategy or the standard protocol provided by the PreOmics iST Kit. Peptide samples were analyzed with the LTQ-Orbitrap mass spectrometer or the Orbitrap Fusion Lumos mass spectrometer, each coupled to an Easy-nLC1000 HPLC pump (Thermo Fisher Scientific). Proteins were identified and quantified using Proteome Discoverer (v2.1, Thermo Fisher Scientific). Proteomics data were analyzed further using the statistical software suite, Qlucore (version 3.3, http://www.qlucore.com/), XINA (21) and the protein-protein interaction network (Supplementary material).

## Results

### Mouse study rationale and design

**Figure 1:**
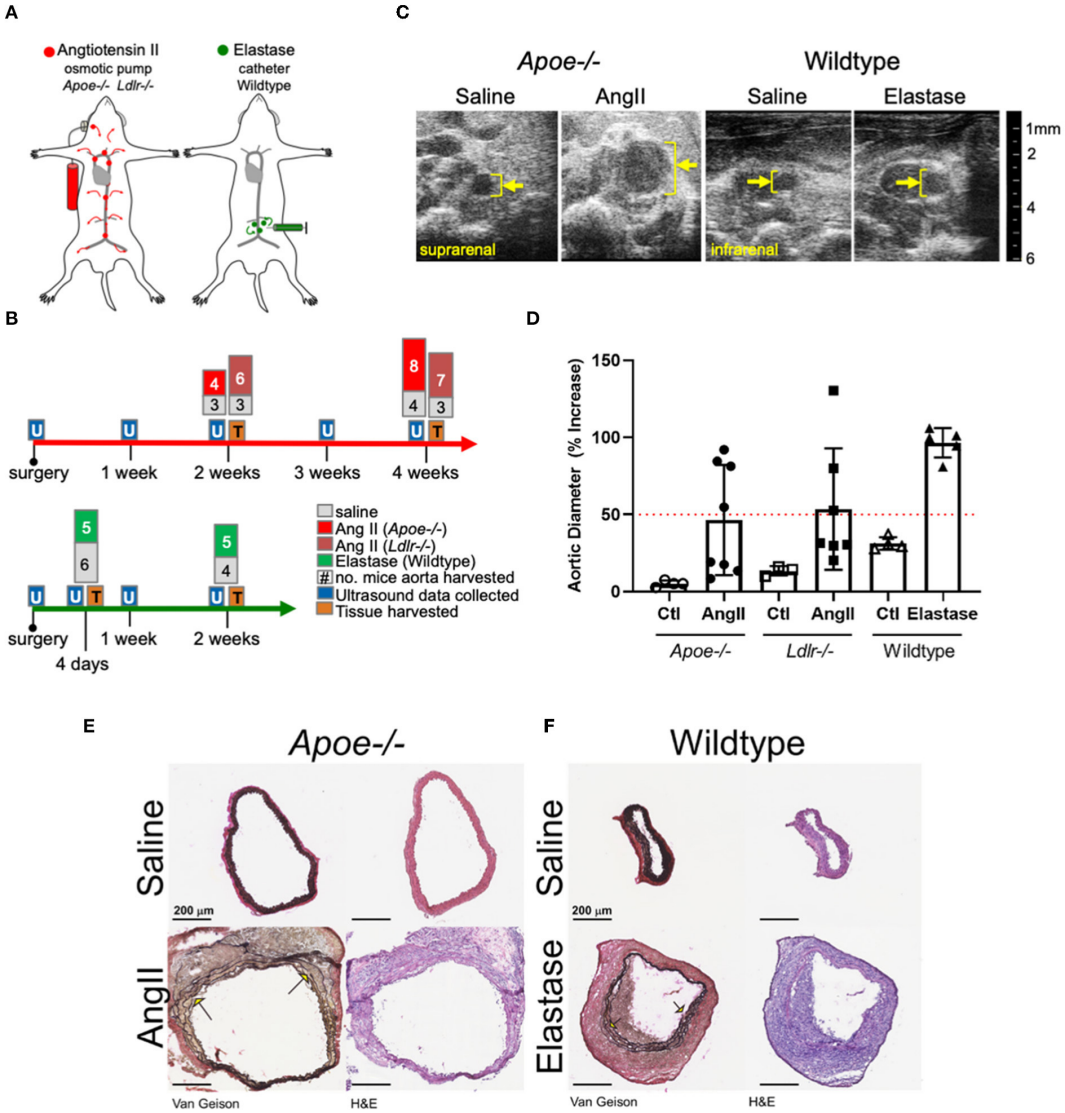
Study design and abdominal aortic aneurysm model phenotypes. **(A)** AngII vs. elastase administration in mice. AngII was infused subcutaneously using an osmotic pump; elastase was administered directly into the infrarenal aortic lumen using a catheter. **(B)** Timeline and experimental design detailing collection of ultrasound data and tissues for proteomics. The number of aortas collected are indicated at tissue collection time points. **(C)** Representative cross-sectional ultrasound images for final time point of each model. Yellow bracket indicates luminal area and expansion. **(D)** Graph depicting percent increase in aortic diameter over baseline ultrasound measurements in each model. *Apoe*^−/−^ represented as circles, *Ldlr*^−/−^ by squares, and elastase wildtype by triangles, with saline controls represented by empty shapes respective to group; red dotted line indicates AAA (50% increase over baseline). **(E,F)** Van Gieson (collagen and elastin) and H&E (nuclear and cytosol) staining of the suprarenal and smaller infrarenal segments of *Apoe*^−/−^ and wildtype aortas, respectively. Arrows identify elastin breaks.

The primary aim of the present study involved identifying and comparing proteins and molecular pathways defining the two most commonly used mouse AAA models: AngII infusion into *Apoe*^−/−^ or *Ldlr*^−/−^ male mice, and the localized, luminal perfusion with elastase into the infrarenal aorta of male wildtype mice (Figure 1A). To capture a proteome correlated with a “developing” aneurysm as well as one that correlated with an “established” aneurysm, we chose two time-intervals for aortic tissue harvest: 2 and 4 weeks after initiation of AngII infusion, and 4 days and 2 weeks after elastase exposure (Figure 1B). The extent of aortic expansion was determined by ultrasound-based measurements of aortic diameter (Figure 1C; Supplementary Table 1), depicted as percent increase at each time interval (Figure 1D). Aortic diameter dilation of >50% (relative to baseline prior to aneurysm induction) was considered aneurysmal. Compared to elastase, expansions due to AngII infusion demonstrated greater variability (Figure 1D), consistent with previous reports (16, 22).

Histopathology demonstrated adventitial thickening, cellular infiltrate, and elastin layer tears or breaks common to both two models (Figures 1E,F). The difference between diameter of representative aortas from saline-infused from each model can be attributed to the suprarenal or infrarenal locality of the AngII and elastase models, respectively.

### Global proteomic profiling of mouse AAAs

**Figure 2:**
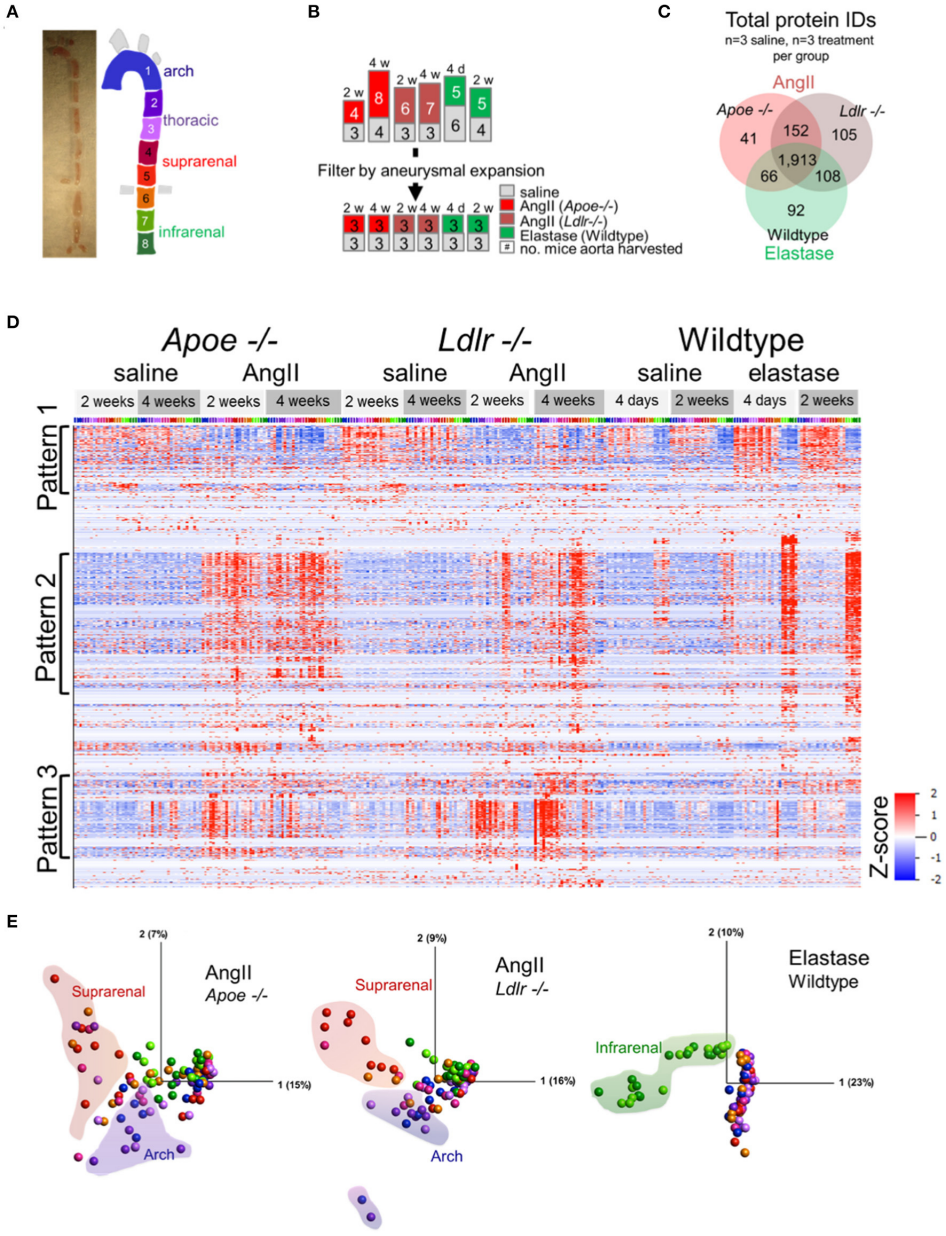
Proteomic profiling of three AAA models. **(A)** Gross image of artery and segmentation method, and cartoon depiction of segments with corresponding numbers and colors. **(B)** Representation of the number of mice included in proteomic analysis (top set of numbers) and filtration of this group by aneurysm severity (bottom set). **(C)** Venn diagrams for the number of proteins, increased or decreased, identified across the three animal models. **(D)** Hierarchical cluster analyses (by protein, rows) showed their relative abundances across models (grouped at top) and segments [colored across top correlated to coloration in **(A)**]. **(E)** Principal component analysis representing protein expression for each artery fragment from each individual model, including saline controls. Coloration of the fractions follows cartoon in **(A)** with shaded outlines indicating general grouping of arterial segments on the plot.

To capture the spatiotemporal protein expression patterns within and across experimental AAAs, we performed label-free proteomic profiling of the arch, thoracic, suprarenal, and infrarenal segments of the aorta (Figure 2A). The proteomics data reflected the variation in the aneurysmal expansion between and within the AAA models (Supplementary Figure 1; all mice studied). This variability, particularly notable in AngII-infused mice, could pose problems if the proteomes of mice with an aortic expansion <50% would dilute the aneurysm-specific variation in the proteome. We therefore selected a subset of mice from each group that would specifically capture the aneurysm-specific proteome, prioritizing the final analyses on data acquired from three mice, per procedure, with the most severe aneurysm pathology (Figure 2B) for subsequent analyses.

### Proteome profiling revealed distinct aortic molecular patterns

A total of 2,477 proteins from all three AAA groups and appropriate controls were quantified (Supplementary Table 2). A Venn diagram demonstrated the interconnectivity of these models (1,913 proteins were shared amongst all three groups), as well as the unique features resulting from the specific aneurysm model and/or the genetic background of each group (Figure 2C). Hierarchical clustering of protein abundances revealed three major patterns (Figure 2D). Pattern 1, a baseline pattern, contains predominately proteins associated with AngII model controls, or segments of artery not directly affected by surgery (i.e., artery segments 1 through 6) in the elastase model. Pattern 2, a general pathology pattern, contains proteins whose expression coincides with aneurysm locality within each model (suprarenal segments in the AngII groups, infrarenal segments in the elastase model); and Pattern 3 is populated by proteins with increased expression in the arch segments of *Apoe*^−/−^ and *Ldlr*^−/−^ aneurysmal mice.

The principal component analyses (PCA, Figure 2E) further delineated the intra-group sources of variability. Segments of the suprarenal and arch aorta revealed the greatest variation in the *Apoe*^−/−^ group, likely due to the aneurysmal and atherosclerotic plaque features, respectively. Several points representing the arch and suprarenal segments remain intermingled, possibly demonstrating the interference of atherosclerotic and aneurysmal features in these mice, even in the absence of a “Western” diet (Figure 2E). The *Ldlr*^−/−^ plot, on the other hand, showed the fewer intermingled suprarenal and arch segment points, possibly due to the expected absence of atherosclerotic plaques in these mice in the absence of a “Western” diet (23). In complete contrast, and as expected for the elastase group in wildtype mice, infrarenal fractions cluster closely together and provide the most notable variation (Figure 2E).

### High-dimensional model-based clustering for spatial resolution of aneurysmal proteins

**Figure 3:**
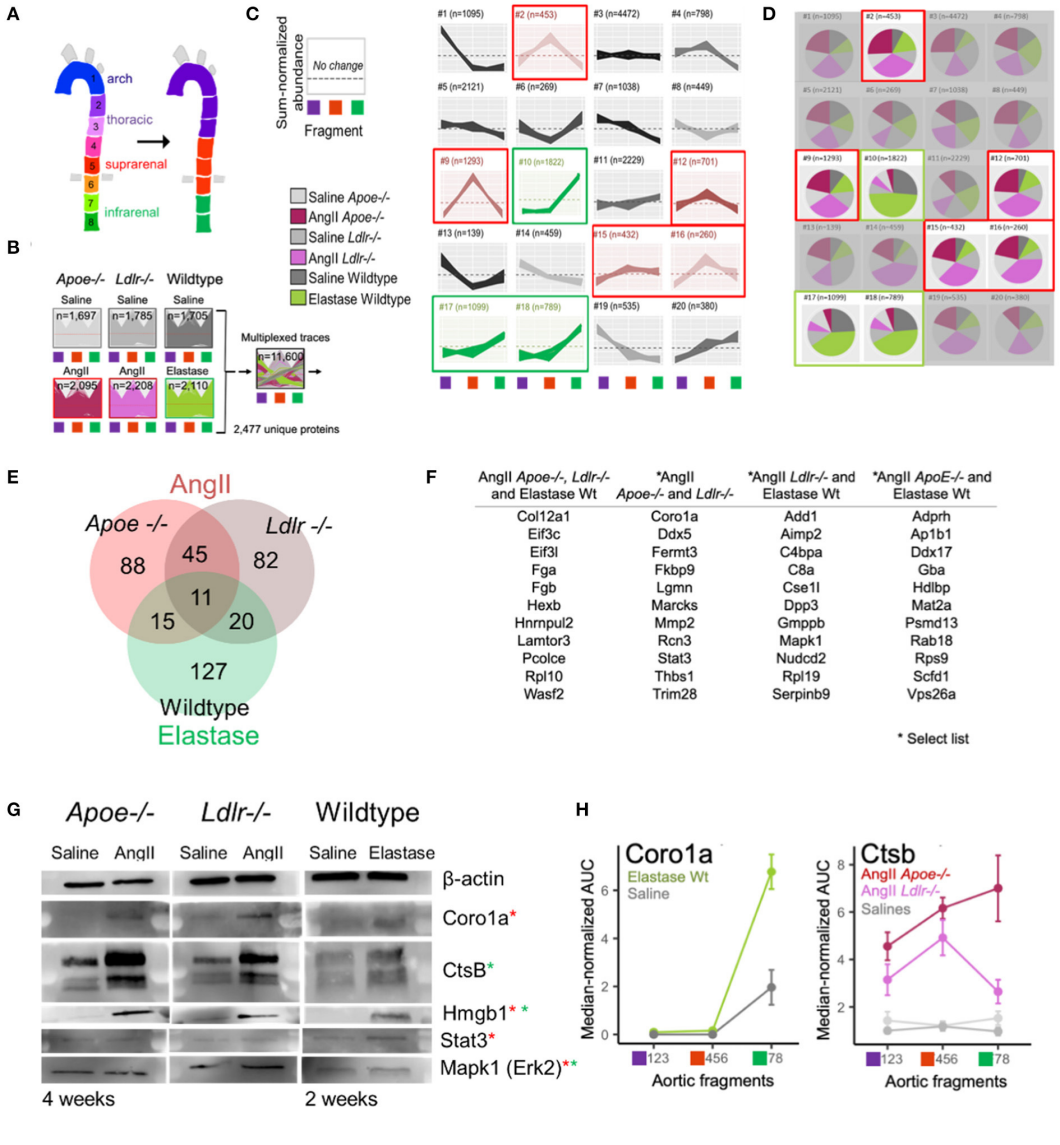
High-dimensional cluster analyses identified AAA model proteins. **(A)** Original eight arterial fragments re-colored to represent three major segment groups for subsequent clustering. **(B)** Protein signatures from all three AAA models were combined to generate a single cluster depicting the variance from the combined protein data. **(C)** Protein abundance clusters showing sum-normalized quantified data on the y-axis and arterial segment group [colored according to scheme in panel **(A)**] on the x-axis. Selected expression patterns colored red (AngII) or green (elastase) according to model. **(D)** Composition pie charts depict source (i.e., model) from which each cluster derives its constituent proteins. Gray shades represent saline groups, dark (*Apoe*^−/−^) and light (*Ldlr*^−/−^) pink represent AngII model, and green represents the elastase-perfused mice. Red (AngII) and green (elastase) outlines mirror highlighted clusters in **(B)**. **(E)** Venn diagram shows the number of proteins identified as being correlated significantly with aneurysm development in each model. **(F)** A select set of proteins unique to one model or shared across two or more models are listed. **(G)** Western blots of candidate aneurysmal proteins. Red and green asterisks indicate from which model(s) the proteins were enriched. **(H)** Coro1a and Ctsb proteins were omitted from elastase and AngII dataset filtering, respectively, due to strict filtering criteria, but still appear to reflect aneurysmal signatures.

The PCA (Figure 2E) revealed that the protein abundance patterns of the eight segments can be simplified into three arterial segments of interest: arch (segments 1–3), suprarenal/thoracic (4–6), and infrarenal (7 and 8) (Figure 3A). Subsequent, in-depth analyses of the data was performed using these data-identified arterial segments. The multi-dimensional nature of our dataset, including model, treatment, time interval, and aortic section, required careful consideration to identify proteins contributing to aneurysm development and progression. Thus, we turned to a high dimensional dataset clustering strategy that combined protein abundance profiles from multiple datasets into a single dataset for clustering (21) (Figure 3B). The single 20-cluster output, comprising a combined 11,600 protein profiles pertaining to 2,477 unique proteins, depicted the variation across all six groups—the three aneurysms and their saline control counterparts (Figures 3B,C). Abundance profiles expected from aneurysm-specific responses for the AngII model (suprarenal, red-highlighted clusters) or the elastase model (infrarenal, green-highlighted clusters) were selected (Figure 3C). The pie charts depict group compositions for each cluster, with red-highlighted, red/pink dominated pie charts demonstrating a dominance of proteins from the AngII groups whereas green-highlighted pie charts show a dominance of proteins from the elastase group (green shades; Figure 3D).

Proteins from the five AngII- and three elastase- dominated clusters were further filtered to identify those with a profile unique to the aneurysm, i.e., the counterpart saline profile did not fall within any of the selected cluster patterns. These filtered protein lists were refined by identifying proteins with a fold change ≥ 2 or ≤0.5 as compared to their localized saline controls, *p*-value ≤ 0.05, and false discovery rate (FDR) ≤ 0.05. This high dimensional clustering and filtering step reduced the proteins of interest to include 159 and 158 proteins in AngII-infused *Apoe*^−/−^ and *Ldlr*^−/−^ mice, respectively, and 173 proteins in the elastase mouse group (Figure 3E; Supplementary Table 3). The elastase model contains 127 unique proteins and shares 31 and 26 proteins with AngII-infused *Ldlr*^−/−^ or *Apoe*^−/−^ mice, respectively; the two groups in the AngII model share 56 common proteins.

The list of eleven proteins shared across all groups includes three proteins of particular interest: regulator complex protein Lamtor 3 (LAMTOR3), procollagen C-endopeptidase enhancer 1 (PCOLCE), and collagen alpha-1 (XII) chain (COL12A) (Figure 3F; Supplementary Table 3). LAMTOR3 participates in mitogen activated kinase 1 (MAPK1/ERK2) signaling (24, 25), a protein studied previously in aneurysm (26, 27) and enriched in AngII *Ldlr*^−/−^ and wildtype elastase aneurysms. PCOLCE interacts with THBS1 (28) and MMPs such as MMP2 (29), two proteins identified in both AngII groups and implicated previously in mouse aneurysm models as well as human aneurysms (26, 27, 30–33). In addition to PCOLCE, the presence of COL12A in all three aneurysm groups indicates a role for fibrosis and/or ECM interactions (34). Other proteins listed in Figure 3F include those with relevance to the networks shown further below. A full list of filtered aneurysmal proteins is presented in Supplementary Table 3.

Western blot analyses and tissue immunostaining validated select proteins (Supplementary Table 4). For the later interval in each model, we readily detected and verified an increase in signal for CORO1A (AngII-filtered protein), CTSB (elastase-filtered) and HMGB1 (AngII and elastase-commonly filtered) across all three groups (Figure 3G). Although the presence of STAT3 and MAPK1 could be confirmed in each model using Western blot analysis, the expression was not increased in aneurysmal tissues as compared to control (Figure 3G). While immunostaining revealed FERMT3/KINDLIN3 to be increased moderately in AngII *Apoe*^−/−^ tissues as compared to control, the difference did not reach significance (Supplementary Figure 2).

Due to the multi-step, stringent filtration process chosen for this proteomics dataset, there are proteins ultimately listed as associated with only one AAA model (e.g., CORO1A in AngII, and CTSB in elastase), which are in actuality increased in the respective aneurysm region for both models. CORO1A increased in elastase-administered tissues but was excluded from the elastase model proteome because corresponding saline-perfused tissues exhibited a similar expression pattern (Figure 3H). AngII-infused *Apoe*^−/−^ mice excluded CTSB due to its elevated expression in both supra- and infra- renal segments (Cluster #11 in Figure 3C). This retrospective examination of the proteomes reflected the pros and cons of any given protein filtering strategy.

### Temporal trends in the aneurysmal proteomes differed within and between AAA models

**Figure 4:**
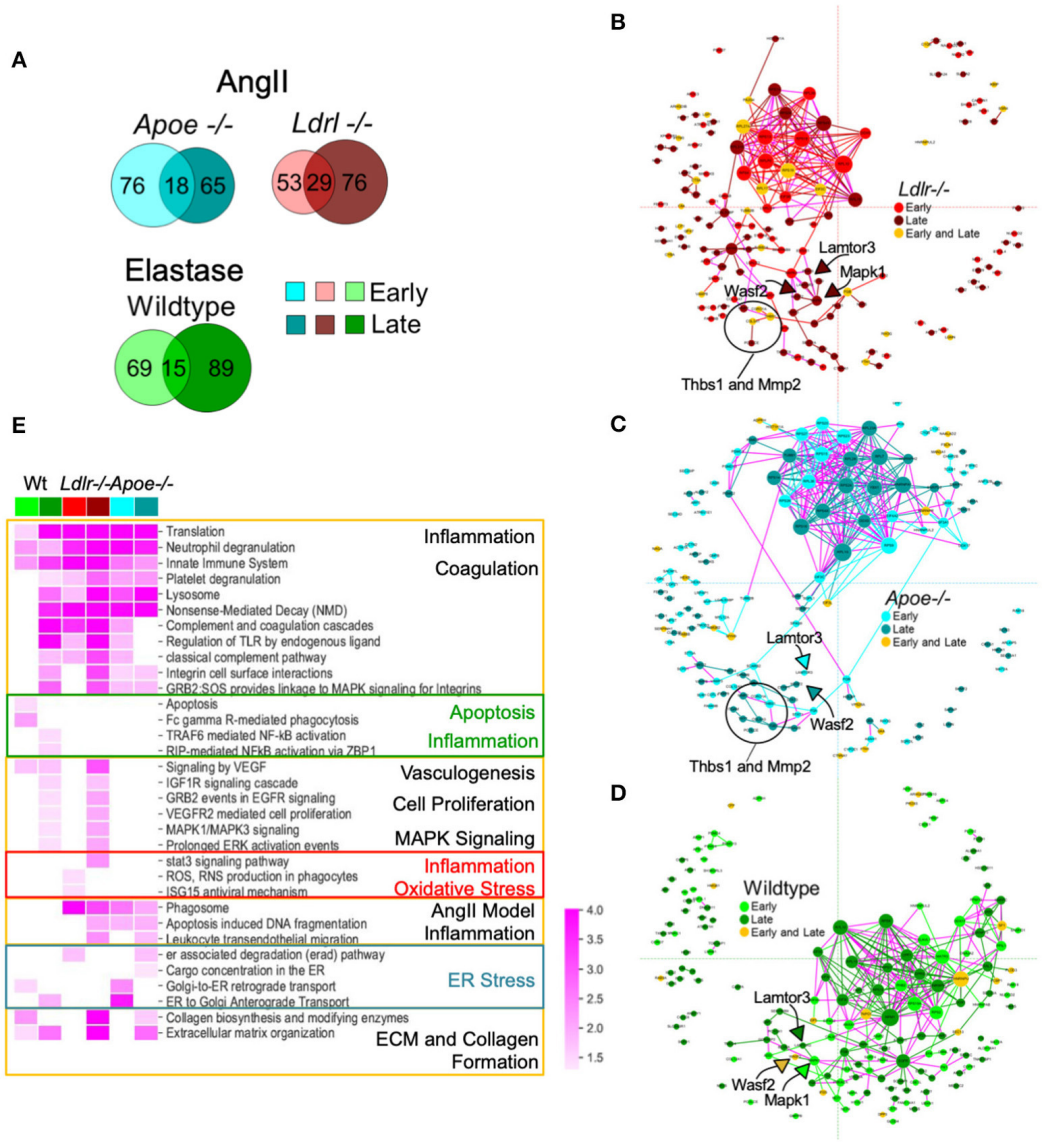
Protein-protein interaction (PPI) networks associated with aneurysmal tissue in each AAA group and model. **(A)** Venn diagrams showing aneurysm-associated proteins group and interval. **(B–D)** A fixed coordinate PPI network depicting described interactions among each AAA model's aneurysm-enriched proteins. When superimposed, proteins shared across the networks occupy the same coordinates. Links connecting early and late interval proteins are represented in purple. Node size is proportional to degree (i.e., the number of connections). **(E)** Heatmap showing the enriched pathways (FDR <0.05) of each mouse model and time interval (early or late). A darker shade indicates higher enrichment while empty cells indicate non-significant (FDR > 0.05) enrichment. Pathways with similar enrichment values are clustered together. The color scheme follows panels **(B–D)**.

Our study aimed to monitor the proteomes of early (developing) and late (established) aneurysms, providing a means to address a potential mechanism and investigate factors in disease progression. Considering each model separately, Venn diagrams provided an overview of aneurysm-related proteins in early and late time intervals (Figure 4A). With the aim to visualize potential relationships across different AAA proteomes and identify the molecular mediators between early and late intervals, we built subnetworks for each model by mapping the temporally labeled (early, late, or both) proteins on a fixed coordinate system on the protein-protein interaction (PPI) network (Figures 4B–D). The PPI network was built from human interaction databases, and thus the human orthologs for proteins are indicated in the subnetworks. Inspecting the connections within each model's subnetwork revealed the following trends: (1) the largest PPI subnetwork for AngII mice comprised ribosomal proteins that primarily associate with the early interval for *Ldlr*^−/−^ and the late interval for *Apoe*^−/−^ (Figures 4B,C); and (2) the largest PPI subnetwork for the elastase model comprises a distinct group of ribosomal proteins that remain connected to EGFR and MAPK subnetworks primarily associated with the late interval (Figure 4D). This MAPK node connected to LAMTOR3 and WASF2, two proteins identified in all three aneurysm groups. These proteins were associated with the late stage/established AAA in AngII *Ldlr*^−/−^, but with early/developing, late, or both stages for AngII *Apoe*^−/−^ and elastase-perfused mice. Other proteins that were relatively close to the MAPK node include THBS1, MMP2 in both AngII groups, and PCOLCE in all three groups (Figures 4B–D). Particularly in the context of the integrins found in proximity to this subnetwork in the late stage AngII groups (ITGAM/CD11b, ITGB2/CD18, ITGA2B, ITGB3) (35–37), THBS1 (30, 38) and WASF2 (39) may have contributed to adhesion and transmigration activities. The overall topology of the fixed coordinate PPI confirmed, as expected, that the AngII *Apoe*^−/−^ and *Ldlr*^−/−^ models share more similarities to each other than to the elastase model; but a closer look at subnetworks demonstrated that peak expression times vary across all three groups.

A representative subset of significantly enriched pathways derived from AAA proteomes at early and late time intervals of each group are presented in a heatmap (Figure 4E; Supplementary Figure 3 for the complete list of pathways). Signaling pathways related to translation and transcription, inflammation and inflammatory cell activities, and coagulation pathways were associated with all three groups, primarily the early and late interval proteomes of both AngII models, and the late interval proteome of the elastase model (Figure 4E). The early interval proteome for the elastase model uniquely associates with apoptosis-related pathways and phagocytosis (Fc gamma R-mediated phagocytosis), while oxidative stress (ROS, RNS production in phagocytes) is identified in the early proteome of the AngII *Ldlr*^−/−^ group. The early and late interval proteome of the AngII *Apoe*^−/−^ group share several features including cell damage and apoptosis (apoptosis-induced DNA fragmentation, phagosome), and features that may indicate endoplasmic reticulum (ER) stress (retrograde and anterograde transport, ER associated degradation, and cargo concentration in the ER) (Supplementary Figure 3; Figure 4E). The late interval proteomes of the AngII model shared pathways related to ECM and collagen formation and leukocyte infiltration, while the late interval of the elastase model included pathways of nuclear factor kappa B (NF-κB)-driven inflammation and shared pathways of vasculogenesis (vascular endothelial growth factor, VEGF), cell proliferation, and mitogen-activated protein kinase (MAPK) signaling with the late interval AngII *Ldlr*^−/−^ proteome (Figure 4E).

### Thrombus did not bias aneurysmal proteome data

The proteomic signal from the thrombus could potentially supersede that of the arterial wall at the site of aneurysm expansion. We used laser capture microdissection (LCM) to separate the thrombus from the surrounding arterial wall for proteomics (Supplementary Figure 4; Supplementary Table 5). From six *Apoe*^−/−^ and three *Ldlr*^−/−^ aneurysm LCM samples, we quantified a total of 2,311 proteins. A two-group comparison between the arterial wall and thrombus proteomes was performed for each genotype (Supplementary Figure 4; Supplementary Table 5). Most differentiating proteins within each group were increased in the arterial wall. Since the wall and thrombus proteomes largely overlap, the thrombus itself did not account for the differences between aneurysm and control tissues.

### Human aneurysmal tissues provided limited but distinct proteome signatures

**Figure 5:**
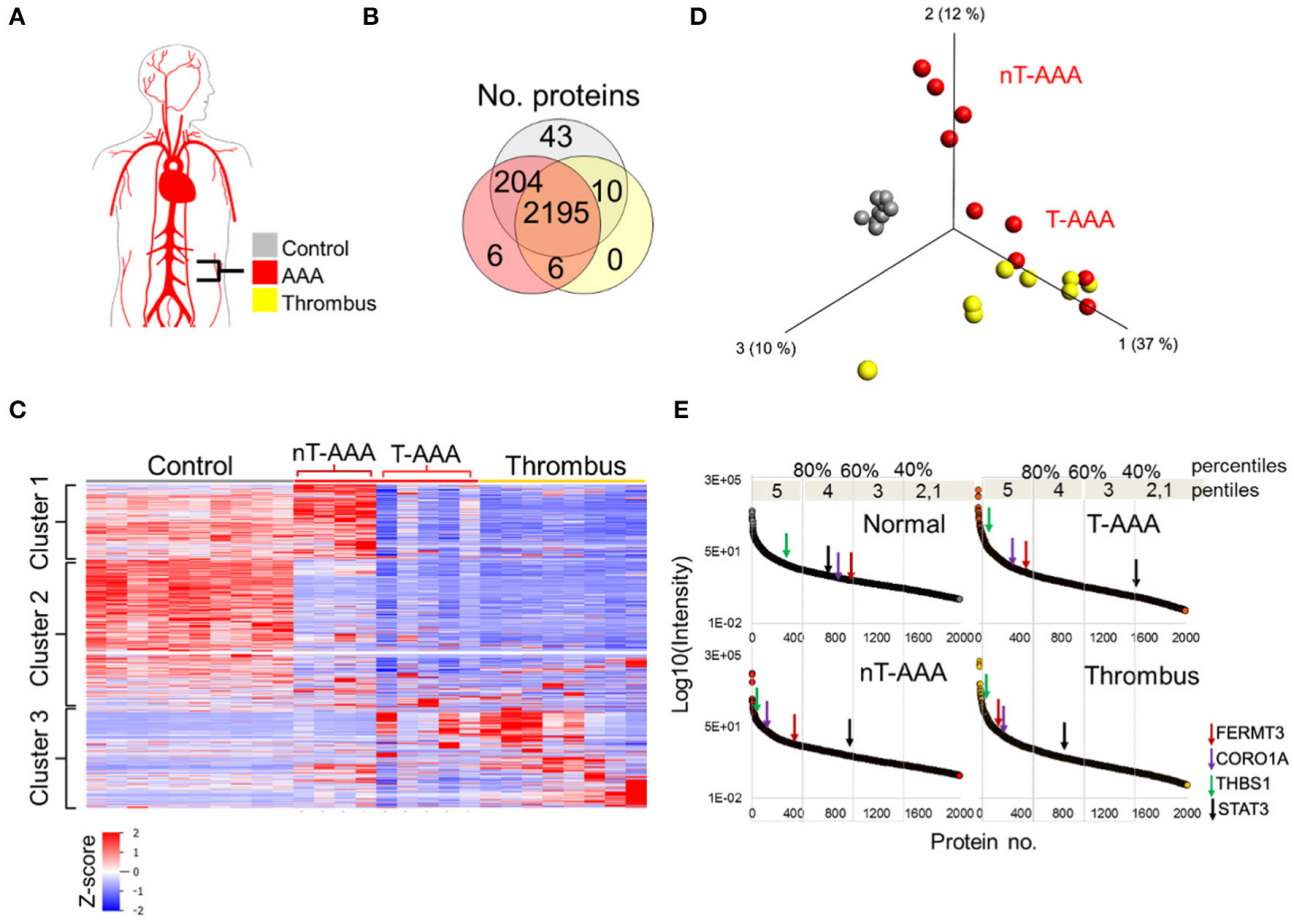
Human aortic aneurysmal tissue proteomics. **(A)** Schematic of aneurysm tissue samples and sample color scheme. **(B)** Venn diagram comparing the number of proteins identified across the three tissue types. **(C)** Hierarchical cluster analysis showing the relative abundances of proteins across the human tissue samples. **(D)** PCA depicting that control samples exhibit a unique proteome whereas some aneurysmal tissues cluster closer to the thrombus samples (T-AAA), but others do not (nT-AAA). **(E)** Protein abundances are ranked from high to low for each tissue sample in order to compare the relative change in abundance of proteins across the samples. The changes in abundance for four selected proteins are shown using colored arrows along each abundance curve.

To further substantiate the clinical relevance of the mouse models, we performed a proteomics study on human diseased tissues. Nine human infrarenal AAA samples were acquired, and eight were separated into thrombus and AAA tissues, leaving one sample with no distinct thrombus as an intact sample. Non-aneurysmal, infrarenal aortic tissues from 10 autopsy cases served as controls (Figure 5A; Supplementary Table 6). A total 2,485 proteins were quantified, however, initial analyses showed control tissues to contribute 43 unique proteins as compared to 6 from AAA tissues and none from thrombus tissues (Figure 5B).

Hierarchical clustering of the protein abundances yielded three major clusters that showed not only a marked difference between the control and aneurysmal tissues, but also a subdivision within the aneurysmal aortic wall samples (Figure 5C). Specifically, five AAA samples clustered closer to the thrombus samples, hence designated thrombus-like AAA (T-AAA), whereas the remaining four aneurysmal aortic wall samples formed a distinct cluster and were designated non-thrombus AAA (nT-AAA, Figure 5D). The heatmap also demonstrated that, when compared to the control tissues, aneurysmal tissues contain a low abundance of most detected proteins, likely reflecting the ECM breakdown, medial degeneration, and acellular composition of these tissues. Only a few proteins comprised the tissue-specific proteome signal, defined primarily by Cluster 1 for nT-AAA samples and Cluster 3 for T-AAA and thrombus samples (Figure 5C). The full list of the protein abundances within each tissue type is represented in Supplementary Table 6.

Since the protein distribution signal varied greatly between control and aneurysmal tissues, we instead compared tissues using a percentile ranking of their averaged protein abundances. Each protein is plotted according to relative abundance within each tissue type, and each plot divided to five percentile ranks (pentiles) in order to identify proteins moving from lower percentile ranks in control tissues to higher percentile ranks in the aneurysmal tissues (Figure 5E). We mapped the locations of some proteins of interest as identified from the animal models such as FERMT3/KINDLIN3, CORO1A, THBS1, and STAT3, and observed interesting trends: FERMT3/KINDLIN3, CORO1A, and THBS1 move up in abundance rank between control and each aneurysmal tissue, with FERMT3/KINDLIN3 and CORO1A moving from the 60th to 80th percentiles, and THBS1 remaining in the upper most pencentile range (Figure 5E). STAT3, on the other hand, moved to lower abundance ranks in all three aneurysmal tissues, dropping to as low as the 40th percentile in the T-AAA tissues.

### Murine AAA models exhibited overlapping and unique features compared to the human disease network

**Figure 6:**
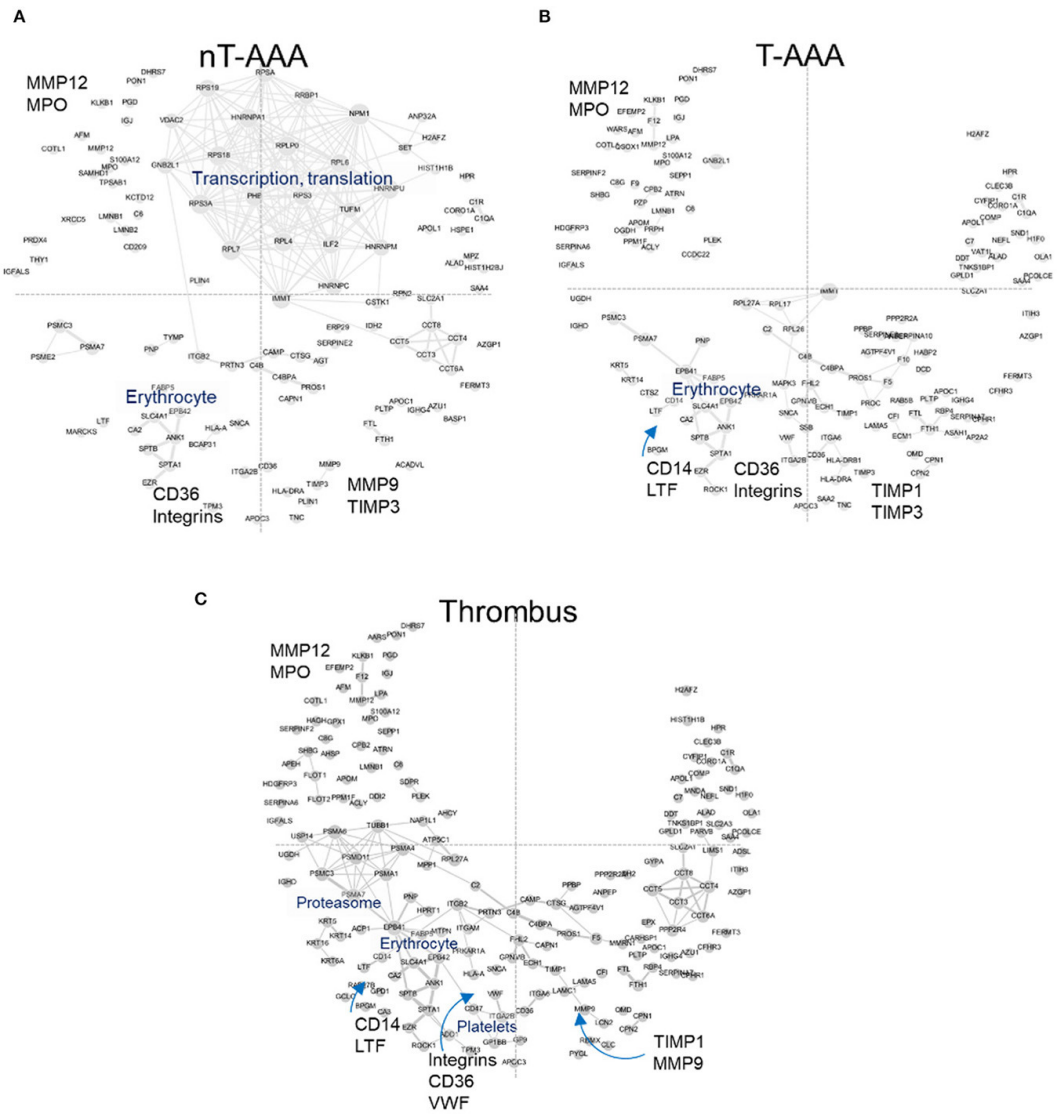
Human aneurysmal tissue networks. **(A–C)** The PPI fixed coordinate networks for proteins enriched in each aneurysmal tissue type.

The increase in abundance rank for FERMT3/KINDLIN3 and CORO1A led to the examination of other proteins exhibiting a similar behavior (Supplementary Table 7). These analyses resulted in 122 (nT-AAA), 160 (T-AAA) and 192 (thrombus) proteins that were mapped to the PPI network (Figure 6). Larger font and arrows are used to highlight several topological features, proteins, and/or subnetworks shared among the three networks. For example, a subnetwork of proteins related to translation and transcription predominates the nT-AAA network (Figure 6A) but remained almost completely absent from the T-AAA and thrombus networks (Figures 6B,C). The latter two networks, while generally comprising a complementary topology, contain notable attributes. Specifically, the thrombus network contained a subnetwork populated by proteasome-related proteins such as proteasome subunit alpha 1 (PSMA1), 4 (PSMA4), 6 (PSMA6), and 7 (PSMA7), which further extends to both an erythrocyte subnetwork and a platelet subnetwork, including scavenger receptor CD36, platelet glycoproteins GP1BB and GP9, von Willebrand factor (VWF) (Figure 6C). The erythrocyte subnetwork, including erythrocyte membrane protein band 4.2 (EPB42) and/or protein 4.1 (EPB41), spectrins beta and alpha 1 (SPTB and SPTA1), and ankyrin-1 (ANK1) is present in each the T-AAA, nT-AAA, and thrombus network (Figures 6A,B). The interactions between this erythrocyte subnetwork and the proteasome and platelet subnetworks specifically in the thrombus PPI network could reflect inflammation and oxidative stress contributed by a thrombus (7, 10, 40, 41). Proteins shared among the three networks include matrix metalloproteinases MMP9 and MMP12, the metalloproteinase inhibitors TIMP1 and TIMP3, and myeloperoxidase (MPO) are identified in at least two the networks, and each protein has been identified previously in at least one proteomics-based aneurysm study (11). In addition, integrins including ITGB2 (CD18), ITGAM (CD11b), and ITGA2B are identified, supporting a role for leukocyte infiltration within aneurysmal tissues (35, 37, 42). The monocyte/macrophage marker CD14 is linked to LTF (lactoferrin) that has been shown to have inflammatory effects on macrophages (43–45).

**Figure 7:**
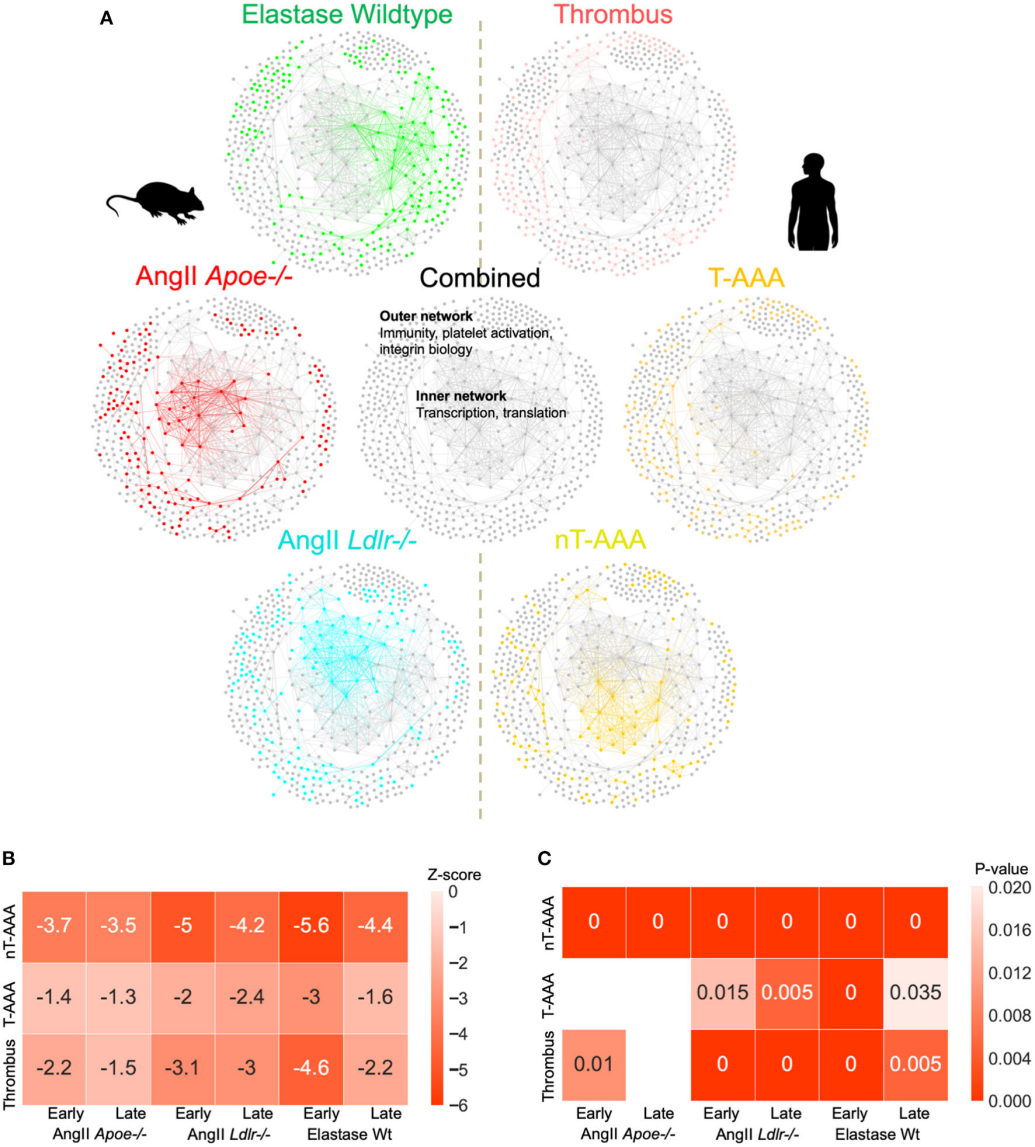
AAA animal model and human tissue consensus network. **(A)** The contribution of each proteome to the combined/consensus network. **(B)** Z-scores of the network closeness between human tissue subnetworks and mouse model subnetworks. A lower z-score (higher absolute values) indicates a higher closeness between the respective pair of subnetworks, suggesting a higher molecular association in the interactome. **(C)** Empirical *p*-values based on 200 randomizations. Heatmap cells annotated as “0” indicate a *p*-value < 0.005. Empty cells indicate insignificant (*p* > 0.05) associations. Z-scores and *p*-values are derived from large numbers of randomized subnetwork instances that were constructed using degree-preserving randomization.

We next combined all three sets of mouse and human aneurysmal proteomes, and created a combined consensus network (Figure 7; Supplementary Figure 5; Supplementary Table 8). The combined network contains a highly interconnected, central subnetwork comprised largely of ribosomal proteins and other transcription/translation-related proteins, and an outer subnetwork that comprises an extensive network of immunity, platelet activation, and integrin biology-related proteins (Figure 7). The proteome of the three mouse model datasets and the human nT-AAA contributed unique and overlapping nodes to the central subnetwork, whereas no contribution by human thrombus and T-AAA proteomes occurs (Figure 7; Supplementary Figures 5E–G). A more general assessment of this combined network leads to the following initial impressions: (1) a prominent deviation between the elastase model and any of the other networks due to its largest subnetwork localized to the right side of the network (Figure 4D); and (2) the consistent presence of inflammation and leukocyte migration-related pathways throughout all six networks.

To assess the degree of association between human tissue and mouse model subnetworks at the molecular level, we performed network closeness analyses (Figures 7B,C). Both analyses depict the highest level of similarity between all three aneurysm model groups and the nT-AAA human tissue type, likely attributed in part to the central transcription/translation subnetwork. In contrast to the human thrombus tissue, which has relatively high overlap with nearly all mouse groups, the disparity between T-AAA and all three mouse groups indicates this tissue type to be relatively unique to the human disease.

## Discussion

**Figure 8:**
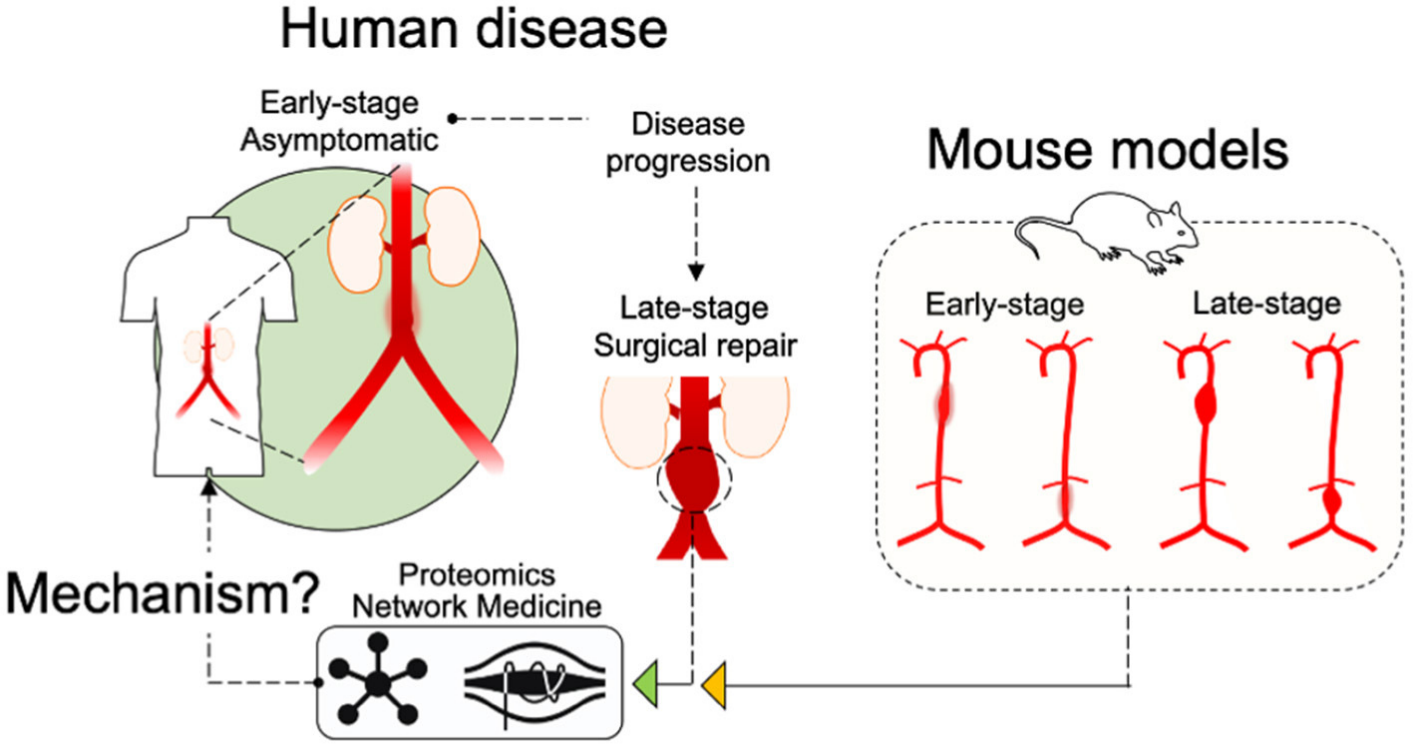
Schematic of study design. An illustration of the study design indicates that “Early-stage” and “Late-stage” tissues from the mouse models, as well as “Late-Stage Surgical repair” human tissues were assessed by Proteomics and Network Medicine to search out common mechanisms.

The use of murine AAA models in research studies has several purposes, including the potential to examine elements of disease development over time. Our study design includes the consideration of temporal features for each group aiming to capture developing and late-stage aneurysmal molecular features. As a consequence, the current study reveals how these models progress and, when considered in context of the human tissue proteome(s), how their progression relates to the human disease (Figure 8). A comparison of “early” and “late” tissue proteomes reveals several consistent elements of diseased tissue in all time intervals and aneurysm groups including translation, inflammation, platelet activity and coagulation, and MAPK signaling. The differences between time intervals provide valuable insight to the progression of each model. Early time interval pathways include indications of apoptosis and phagocytosis (elastase *wildtype* and AngII-infused *Apoe*^−/−^) and oxidative stress (AngII-infused *Ldlr*^−/−^). The late time interval proteome is populated by pro-fibrotic and leukocyte infiltration pathways (AngII model), angiogenesis, cell proliferation, and MAPK signaling (elastase wildtype and AngII *Ldlr*^−/−^), and NF-κB-mediated inflammation (elastase wildtype). Interestingly, both the early and the late proteome of the AngII model include pathways potentially related to ER stress, a feature shown to influence aneurysm (46, 47). The late proteome of each model appears at least partially influenced by injury response(s) to tissue damage at the early stages of aneurysm development. Notably, continued AngII infusion past 28 days can lead to rupture (48), whereas elastase-induced aneurysm expansion ceases by about 14 days without a secondary treatment (49). Continued expansion in this modified elastase model is attributed to chronic inflammation, highlighting a role for leukocyte infiltration (identified in late-interval AngII model proteomes) as a driver of continued expansion in AngII aneurysms (48).

The choice to include only male mice in this study was made primarily to adhere to convention of the field with the hope of producing a dataset applicable to the largest number of studies. Existing evidence suggests that aneurysms produced in female mice are significantly different from those produced in male mice (50). Future studies may further identify the precise differences between the sexes in the context of these mouse models.

Comparison between the human and mouse aneurysmal proteomes in the next stage of this study permitted us to explore the extent of molecular conservation between the human and mouse aneurysmal tissues. The PPI networks of the human aneurysmal tissues indicated many platelet- and acute inflammatory phase-related proteins for the T-AAA tissues; whereas the nT-AAA tissue network was defined by a prevalent subnetwork related primarily to transcription, translation, and protein processing. This heavily interconnected subnetwork comprised the inner network of the combined murine-human AAA PPI network (Figure 7), and the shared nature of this inner network may suggest its relationship to the vascular cells present in these tissue types as compared to the relatively decellularized nature of the thrombus-related tissues in the human samples (51). PCOLCE, CORO1A, and FERMT3/KINDLIN3 in the combined networks suggest a role for collagen turnover and angiogenesis (28, 52, 53), platelet function (54), and integrin biology (54–57) pathways also identified in the murine aneurysm proteomes. The prominence of neutrophil-related proteins and the presence of both MMP9 and MMP12 in the human networks supports the role for sustained influx of damaging inflammatory cell types in human tissues (58), a feature mirrored in the late interval AngII infusion models. A similar conclusion was reached in a study examining the AngII *Apoe*^−/−^ model, the elastase model, and human tissues through the use of genomics (59). Findings in that study led those authors to conclude, in part, that the AngII model likely provides a more “chronic” disease state with continued inflammatory cell infiltrate, while the elastase model eventually reaches a “healing” stage in which fibrosis occurs. Interestingly, despite several study design differences, several of our pathways of interest converge with these previous studies including leukocyte motility, integrin signaling, and THBS1 activity.

The previously described platelet and erythrocyte subnetwork within the thrombus-related human tissues may contribute to the continued inflammatory response, specifically through accumulation of leukocytes, platelets, and erythrocytes, oxidative stress, and the activity of proteolytic pathways (40, 51). The laser capture microdissection component of our study demonstrated that the extramural thrombus found in the AngII model has little or no proteomic difference to the surrounding arterial wall, whereas our proteomic assessment of the human tissue demonstrates differences between the thrombotic and non-thrombotic AAA tissues. The isolation of the AngII-induced thrombus in the extravascular space, as compared to the constant exposure to blood experienced by the human ILT, likely drives this difference. While this evidence does not necessarily determine the thrombus present in murine models to be biologically inert, it lends support to the idea that the thrombus of human AAA holds a significant role in driving the disease (10, 40, 47, 51, 60).

An in-depth exploration of each pathway of interest and its constituent proteins is beyond the scope of this study, however, the study does provide a valuable road map for further analyses. The study provides insight to the ways in which the experimental mouse models represent and/or deviate from the human disease they represent. While we demonstrate that the most common models share several specific proteins and protein pathways with the human disease, the data also reveal interesting details: (1) in contrast to the human ILT, AngII aneurysm-associated, extraluminal thrombus does not appear to contribute to aneurysmal expansion; (2) although the proteomes of the two mouse aneurysm models indicate similar signaling pathways, differences emerging at the late interval proteome point toward events including continued leukocyte infiltration and ER stress setting the stage for continued aneurysmal expansion in the AngII model. Identifying the specific proteins or signaling pathways underlying aneurysm expansion may provide key targets for therapeutic strategies.

## Data availability statement

The authors acknowledge that the data presented in this study must be deposited and made publicly available in an acceptable repository, prior to publication. Frontiers cannot accept a article that does not adhere to our open data policies. Data are available *via* ProteomeXchange with identifier PXD032293.

## Ethics statement

The studies involving human participants were reviewed and approved by University of Rochester Cardiovascular Tissue Bank Internal Review Board. The patients/participants provided their written informed consent to participate in this study. The animal study was reviewed and approved by the Institutional Animal Care and Use Committee of the Beth Israel Deaconess Medical Center.

## Author contributions

SM contributed to the study design, performed most *in vitro* and *in vivo* experiments and data analysis, and drafted and finalized the manuscript. LL and AH conducted bioinformatics and network analyses. JN assisted in *in vitro* experiments and histological assays. HH performed mass spectrometry. AHH and JW performed histological analysis. AD and PL provided advice and critically read the manuscript. SC and DM provided human AAA samples. EA supervised histological assays, provided advice, and critically read the manuscript. AO contributed to scientific discussions, provided human AAA samples, and critically read the manuscript. SS contributed to the study design, supervised mass spectrometry-assisted proteomics and its data analysis, and critically read and finalized the manuscript. MA directed the project, contributed to the study design and data interpretation, critically read and finalized the manuscript, and was responsible for funding and overall administration. All authors contributed to the article and approved the submitted version.

## Funding

This study was primarily supported by a research grant from Kowa Company, Ltd., Nagoya, Japan (to MA), which was not involved in the study design, experiments, data acquisition, and preparation of the manuscript, and in part by the grants from the National Heart, Lung, and Blood Institute (R01HL126901 and R01HL149302 to MA; R01HL136431, R01HL141917, and R01HL147095 to EA; R01HL080472 and R01HL134892 to PL; R01HL133723 and R35HL155649 to AD; R99HL116786 and R01HL147171 to AO; and R01HL158801 to SC).

## Conflict of interest

Author HH is an employee of Kowa and was visiting scientist at Brigham and Women's Hospital when the study was conducted. Author MA has received research grants from Pfizer and Sanofi. Author PL is an unpaid consultant to or involved in clinical trials for Amgen, AstraZeneca, Esperion Therapeutics, Ionis Pharmaceuticals, Kowa, Novartis, Pfizer, Sanofi-Regeneron, and XBiotech, is a member of scientific advisory board for Amgen, Corvidia Therapeutics, DalCor Pharmaceuticals, IFM Therapeutics, Kowa, Olatec Therapeutics, Medimmune, Novartis, and XBiotech, and serves on the Board of XBiotech. The laboratory of PL has received research funding in the last 2 years from Novartis. Author PL has a financial interest in Xbiotech. His interests were reviewed and are managed by Brigham and Women's Hospital and Partners HealthCare in accordance with their conflict of interest policies. The remaining authors declare that the research was conducted in the absence of any commercial or financial relationships that could be construed as a potential conflict of interest.

## Publisher's note

All claims expressed in this article are solely those of the authors and do not necessarily represent those of their affiliated organizations, or those of the publisher, the editors and the reviewers. Any product that may be evaluated in this article, or claim that may be made by its manufacturer, is not guaranteed or endorsed by the publisher.

## Supplementary material

Supplementary Table 1 Raw ultrasound measurements.

Supplementary Table 2 Median normalized, label-free based quantification of the two AAA mouse models and their saline counterparts: AngII (*ApoE-/-* and *Ldlr-/-*) and elastase (wildtype *C57BL/6J*).

Supplementary Table 3 AAA-induced suprarenal and infrarenal filtered proteins.

Supplementary Table 4 Antibodies for Western blot and immunostaining.

Supplementary Table 5 Laser capture microdissection of thrombus and arterial wall tissues in AngII-infused *Apoe*^−/−^ and *Ldlr*^−/−^ mice.

Supplementary Table 6 Label-free based quantification of human aneurysm and control group artery samples.

Supplementary Table 7 Control and aneurysmal protein abundance rank comparisons.

Supplementary Table 8 Mouse and Human Uniprot and MGI IDs.

Supplementary Figure 1 Proteomic profiling of three aneurysm models with all mice included.

Supplementary Figure 2 Fermt3/Kindlin3 immunostaining. **(A)** Representative images of Fermt3/Kindlin3 immunostaining in abdominal aortic cross-sections from saline- and AngII-infused *Apoe*^−/−^ mice. Scale = 300 μm. **(B)** Quantification of Fermt3/Kindlin3 immunostaining in tissue sections from saline (*n* = 3) and AngII (*n* = 3)-infused *Apoe*^−/−^ mice. **(C)** Representative image of an aneurysmal cross-section from an *Apoe*^−/−^ mouse; Adventitia (A), media (M) and intima (I). Scale = 100 μm. **(D)** Representative tissue section of a thrombotic region from an *Apoe*^−/−^ mouse. Scale = 100 μm.

Supplementary Figure 3 Extended heatmap of enriched pathways.

Supplementary Figure 4 Proteome profiling of LCM aneurysmal tissues from AngII-infused mice. **(A)** LCM was performed on aortic segments of 4 and 5 *Apoe*^−/−^ and *Ldlr*^−/−^ mice, respectively. **(B)** Example LCM samples prepared from proteomics. **(C,D)** Heatmap of a two-group comparison between thrombus and arterial wall samples. Protein lists are provided in Supplementary Table 5.

Supplementary Figure 5 High-resolution, node-readable consensus networks with animal and human tissue superimpositions. **(A)** Consensus; **(B)** Elastase wildtype; **(C)** AngII *Apoe*^−/−^; **(D)** AngII *Ldlr*^−/−^; **(E)** Human nT-AAA *Apoe*^−/−^; **(F)** Human T-AAA; **(G)** Human thrombus.
