# Supplementary figures and images for: Identifying novel mechanisms of abdominal aortic aneurysm via unbiased proteomics and systems biology
Source: Front Cardiovasc Med. 2022 Aug 3;9:889994. doi: 10.3389/fcvm.2022.889994 (PMC9382335; doi:10.3389/fcvm.2022.889994)

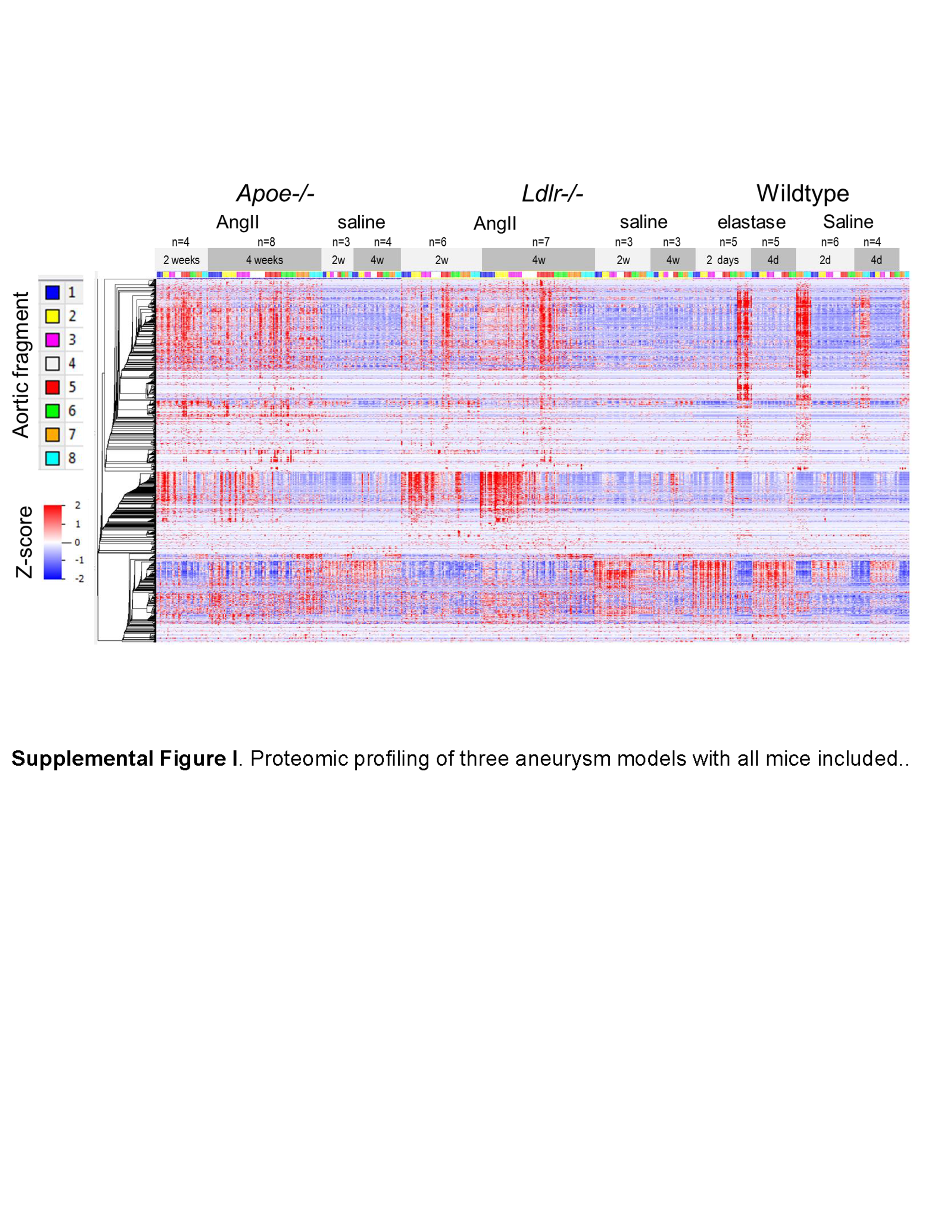

Supplement: Supplementary Figure 1 — Proteomic profiling of three aneurysm models with all mice included. [file Image_1.TIFF]

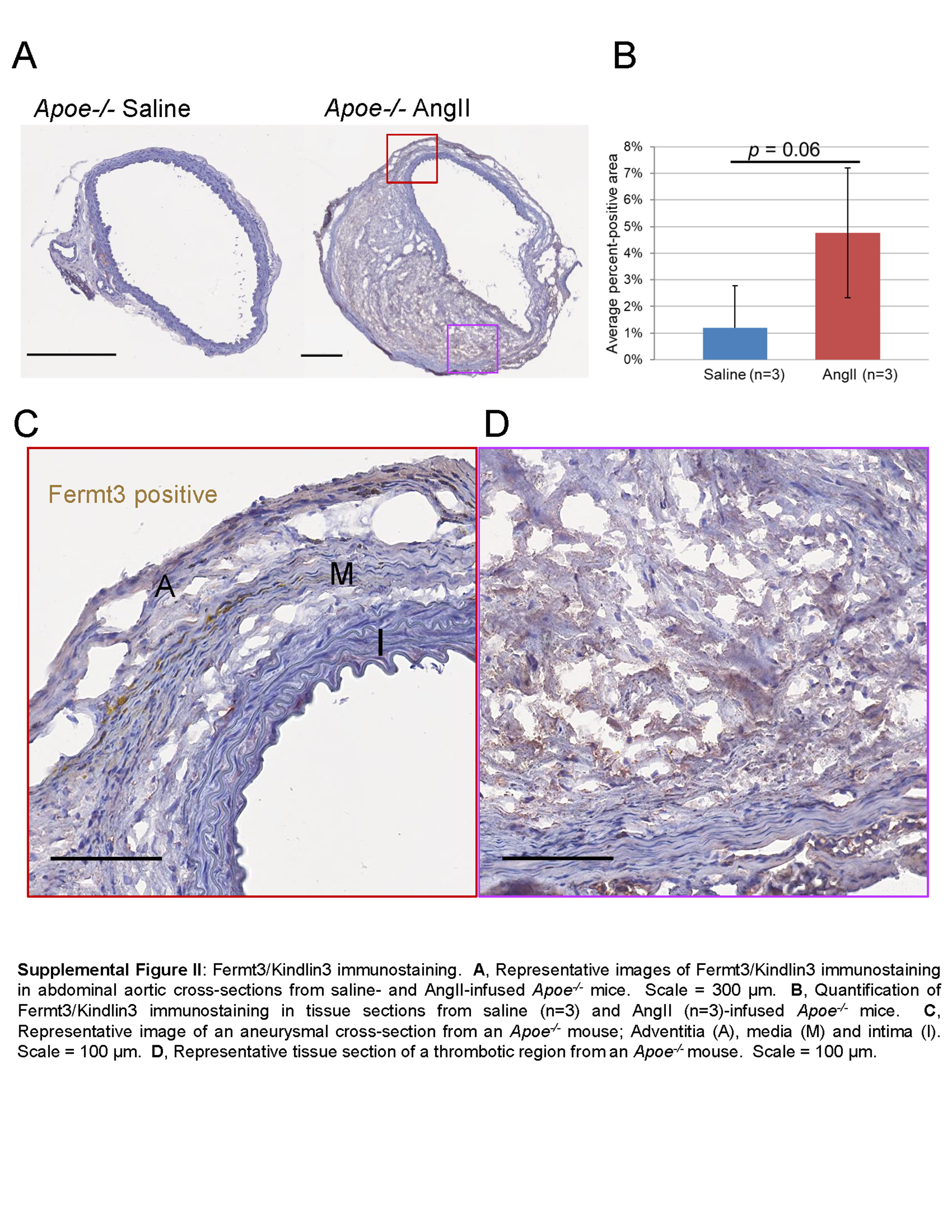

Supplement: Supplementary Figure 2 — Fermt3/Kindlin3 immunostaining. (A) Representative images of Fermt3/Kindlin3 immunostaining in abdominal aortic cross-sections from saline- and AngII-infused Apoe−/− mice. Scale = 300 μm. (B) Quantification of Fermt3/Kindlin3 immunostaining in tissue sections from saline (n = 3) and AngII (n = 3)-infused Apoe−/− mice. (C) Representative image of an aneurysmal cross-section from an Apoe−/− mouse; Adventitia (A), media (M) and intima (I). Scale = 100 μm. (D) Representative tissue section of a thrombotic region from an Apoe−/− mouse. Scale = 100 μm. [file Image_2.TIFF]

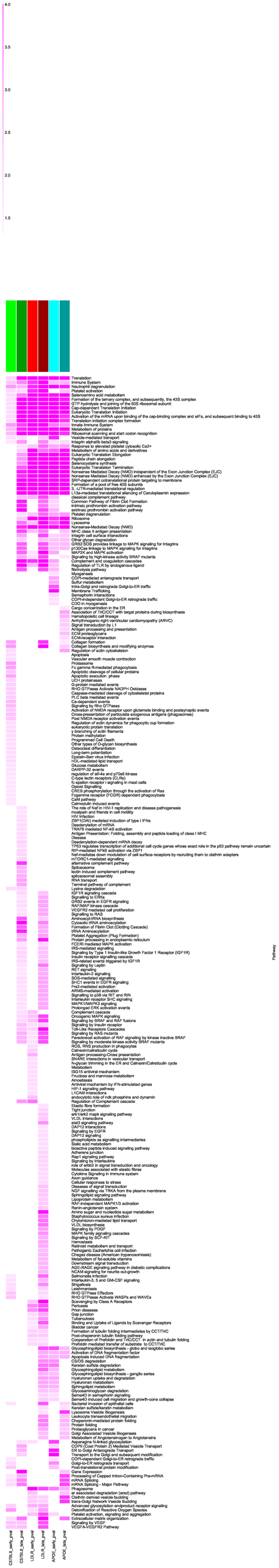

Supplement: Supplementary Figure 3 — Extended heatmap of enriched pathways. [file Image_3.TIFF]

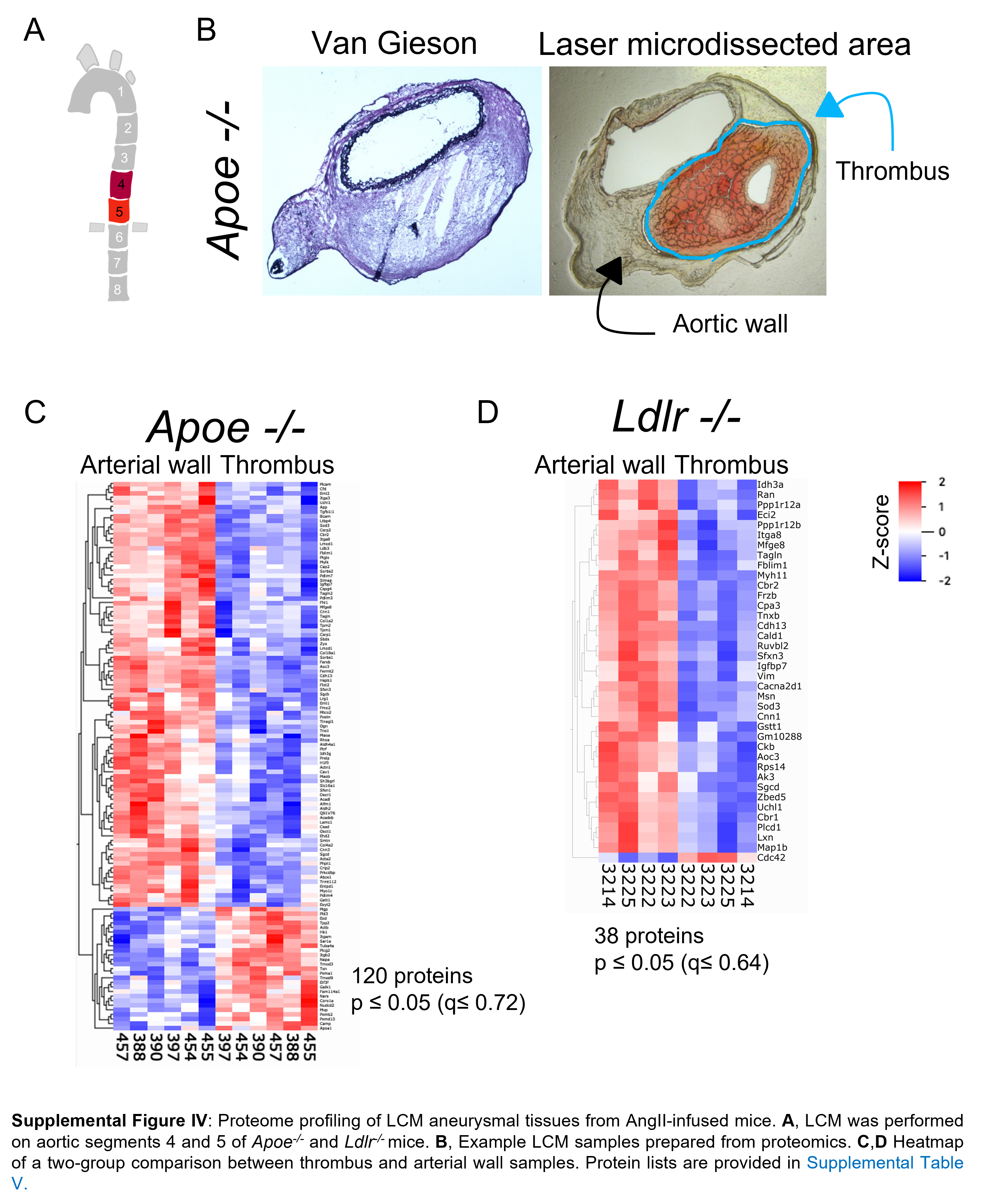

Supplement: Supplementary Figure 4 — Proteome profiling of LCM aneurysmal tissues from AngII-infused mice. (A) LCM was performed on aortic segments of 4 and 5 Apoe−/− and Ldlr−/− mice, respectively. (B) Example LCM samples prepared from proteomics. (C,D) Heatmap of a two-group comparison between thrombus and arterial wall samples. Protein lists are provided in Supplementary Table 5. [file Image_4.tif]
